# Differences in Benzene Concentration Across Packaging Types During Normal Use of Benzoyl Peroxide Products

**DOI:** 10.1111/jocd.70470

**Published:** 2025-09-22

**Authors:** Benjamin Xi En Ho, Wen‐Tsao Ho, Yan‐Rung Duh

**Affiliations:** ^1^ Asia American International Academy New Taipei city Taiwan; ^2^ Department of Dermatology Ho wen Tsao Skin Clinic New Taipei city Taiwan

**Keywords:** benzene, benzoyl peroxide, packaging types

Dear Editor,

Benzoyl peroxide (BPO) is a common treatment for acne, but concerns have been raised about the potential formation of benzene in BPO products under varying environmental conditions [1]. This issue has gained attention in Taiwan, where local media have reported on benzene‐related risks, raising public concern. In Taiwanese medical institutes, especially in clinics, patients are given tubes or dispensed ointment boxes. We hypothesized that differences in benzoyl peroxide packaging types, specifically tubes and dispensed ointment boxes, may influence benzene accumulation due to varying levels of air exposure. Previous studies suggest that high temperatures may promote benzene formation in benzoyl peroxide products, potentially increasing health risks due to benzene's known carcinogenicity [2]. Analysis of FAERS data identified skin reactions like irritation but no strong evidence of malignancy, reinforcing the safety of routine use [3]. However, there is a significant need to let people understand about the risks of benzene formation. Research also suggests that adjusting formulations, such as incorporating antioxidants, can significantly lower benzene levels in BPO products, providing a proactive solution for manufacturers to adopt [4].

This experiment aimed to evaluate benzene concentrations in benzoyl peroxide (BPO) products under two temperature conditions—room temperature (approximately 25°C) and high temperature (40°C–70°C)—as the primary variable, with air exposure as the secondary variable. The high‐temperature range was selected to simulate conditions such as a car interior in Taiwan during summer, based on environmental studies. Benzene concentrations were measured in Aczo Gel 50 mg/g (Sinphar) packaged in tubes (10 g) and dispensed ointment boxes (5 g) at room and high temperatures after the 2nd, 4th, and 6th weeks. The high‐temperature group was placed in a heater set to 40°C–70°C. All containers were opened for 10 s twice‐daily to mimic normal use (twice‐daily application). Samples were analyzed in a private laboratory using established gas chromatography methods [1].

The final data of the experiment are shown in the table.

**Table:** 

Benzene concentration	2 weeks later	4 weeks later	6 weeks later
Dispense ointment box at room temperature.	4.09 ppm	5.65	1.51
Tube in room temperature.	23.75 ppm	4.73	4.35
Dispense ointment box at High temperature.	102.65 ppm	49.98	74.86
Tube in high temperature.	518.89 ppm	2125.68	108.85

As the result demonstrated, the concentration of benzene will increase as temperature was higher accordingly in the two groups. In fact, the concentration of benzene shows higher in the tube than the dispensed ointment box (Table). Furthermore, we speculated that the difference in contact area with air influenced the evaporation of benzene, as larger surface area increases the likelihood of release. In addition, we found that in the 6th week, it shows that the benzene production of BPO enormously decreased, it might suggest that the chemical reaction of benzene production had reached the peak. Unfortunately, the sample is not enough, which means it might require more examples to support this hypothesis.

Given these findings, ointment boxes might be a safer choice for patients due to lower benzene production compared with tubes, aligning with Taiwan's dispensing preferences. For all intents and purposes, in our perspective, we think using dispensed ointment boxes provides a safer environment to the patients, due to the lower production of benzene.

## Conflicts of Interest

The authors declare no conflicts of interest.

## Data Availability

The authors have nothing to report.
